# Ewe breed differences in the cervical transcriptome at the follicular phase of a synchronised oestrous cycle

**DOI:** 10.1186/s12864-022-08603-8

**Published:** 2022-05-11

**Authors:** Laura Abril-Parreño, Kieran G. Meade, Anette Kristine Krogenæs, Xavier Druart, Paul Cormican, Sean Fair

**Affiliations:** 1 Laboratory of Animal Reproduction, Department of Biological Sciences, School of Natural Sciences, Biomaterials Research Cluster, Bernal Institute, Faculty of Science and Engineering, University of Limerick, Limerick, Ireland; 2 School of Agriculture and Food Science, University College Dublin, Belfield, Dublin 4, Ireland; 3 Faculty of Veterinary Medicine, Norwegian University of Life Sciences, Ås, Norway; 4 UMR 6175 INRA, CNRS-Université de Tours-Haras Nationaux, Station de Physiologie de la Reproduction et des Comportements Institut National de la Recherche Agronomique, Nouzilly, France; 5 Animal & Bioscience Research Department, Animal & Grassland Research and Innovation Centre, Teagasc, Grange, Co, Meath, Ireland

**Keywords:** RNA-sequencing, Ovine, Cervix, Oestrous synchronisation

## Abstract

**Background:**

Cervical artificial insemination (AI) with frozen-thawed semen results in unacceptably low pregnancy rates internationally. The exception is in Norway, where vaginal deposition of frozen-thawed semen to a natural oestrous routinely yields pregnancy rates in excess of 70%. Previous studies by our group has demonstrated that this is due to differences in cervical sperm transport. However, a potentially important contributory factor is that ewes are inseminated to a natural oestrous in Norway but to a synchronised oestrous across most of the rest of the world. In this study, we interrogated the gene expression of the sheep cervix of four ewe breeds with known differences in pregnancy rates following cervical AI using frozen-thawed semen under the effect of exogenous hormones to synchronise the oestrous cycle. These four ewe breeds (*n* = 8 to 11 ewes per breed) are from two countries: Ireland (Belclare and Suffolk; medium and low fertility, respectively) and Norway (Norwegian White Sheep (NWS) and Fur; both with high fertility compared to the Irish ewe breeds).

**Results:**

RNA extracted from cervical biopsies collected from these breeds was analysed by RNA-sequencing and differential gene expression analysis. Using the low-fertility Suffolk breed as a reference level; 27, 1827 and 2641 genes were differentially expressed in Belclare, Fur and NWS ewes, respectively (*P* <  0.05 and FC > 1.5). Gene ontology (GO) analysis revealed that Fur and NWS had an up-regulation of enriched pathways involved in muscle contraction and development compared to Suffolk. However, there was a down-regulation of the immune response pathway in NWS compared to Suffolk. In addition, GO analysis showed similar expression patterns involved in muscle contraction, extracellular matrix (ECM) development and cell-cell junction in both Norwegian ewe breeds, which differed to the Irish ewe breeds.

**Conclusions:**

This novel study has identified a number of conserved and breed-specific biological processes under the effect of oestrous synchronisation that may impact cervical sperm transport during the follicular phase of the reproductive cycle.

**Supplementary Information:**

The online version contains supplementary material available at 10.1186/s12864-022-08603-8.

## Background

In sheep, low pregnancy rates following cervical AI using frozen-thawed semen is a worldwide problem for the industry (See review by Fair et al. (2019) [1]). The exception is Norway where farmers themselves perform shot-in-the-dark (vaginal) AI with frozen-thawed semen to a natural oestrous and routinely achieve pregnancy rates in excess of 70% [2]. The reason for the success in Norway has been the focus of a number of studies by our group [3–5] and we have identified the breed of the ewe used in Norway to be the reason for the high fertility. Specifically, we have demonstrated that it is the failure of frozen-thawed sperm to traverse the cervix in some breeds compared to others [6]. Recent studies by our group have shown no relationship between gross cervical anatomy (cervical length, number of cervical rings and the appearance of the external *os*), mucus properties (volume and viscosity) and fertility across six European ewe breeds (Norwegian, French and Irish) [7]. Thus, subtler molecular differences in the cervix or its secretions may explain the differences in frozen-thawed sperm transport.

A factor to consider is that ewes are inseminated to a natural oestrous in Norway but to a synchronised oestrous across most of the rest of the world. The use of exogenous hormones for oestrous synchronisation allows farmers increased reproductive and labour efficiency by controlling the timing of ovulation. However, this has been associated with reduced fertility [8]. There are several protocols used for oestrous synchronisation in ewes, but the most widely used protocols are the intravaginal sponges impregnated with progestogen for 12 to 14 days [9]. Several studies have reported negative effects on pregnancy rates [8] and sperm transport in the reproductive tract of the ewe [10] compared to ewes at the natural oestrous cycle. Furthermore, Manes et al. 2014 [11] reported that the presence of intravaginal sponges decreased the conception rates in ewes following cervical AI using fresh semen. Mitchell et al. 2005 [12] observed increased neutrophil recruitment in the presence of an intravaginal sponge, which could be a factor compromising sperm transit across the cervix.

There are contradictory results regarding the effect of oestrous synchronisation on mucus production, with quantities reportedly increasing [7, 13, 14] and decreasing [15] after hormone use. Maddison et al. 2016 [16] demonstrated an increase in mucus production, which was accompanied by an increase in mucus protein composition in Merino ewes after superovulation compared to the naturally cycling ewes. In addition, many proteins were differentially increased or decreased compared to the levels of those proteins at a natural oestrous cycle [17]. These changes in the cervical mucus have a negative impact on sperm viability [18] and only sperm with normal morphology and motility make it into the uterus [19].

Given that the aforementioned ewe breed differences in sperm transport across the cervix are likely due to subtle molecular differences in the cervix or its secretions and the fact that cervical AI is performed to a natural oestrous in Norway but to a synchronised oestrous across the rest of the world. We undertook an RNA-sequencing analysis to interrogate cervical gene expression in these ewe breeds at the synchronised cycle following our previous gene expression analysis of the same ewe breeds at the natural oestrous cycle [20] . Therefore, the aim of this study was to profile the transcriptome of the cervix in two Norwegian and two Irish ewe breeds, with known differences in pregnancy rates following cervical AI with frozen-thawed semen, at the follicular phase of a synchronised oestrous cycle.

## Results

### Clustering of Norwegian against Irish ewe breeds showed an evident divergence in gene expression profiles

**Fig. 1:**
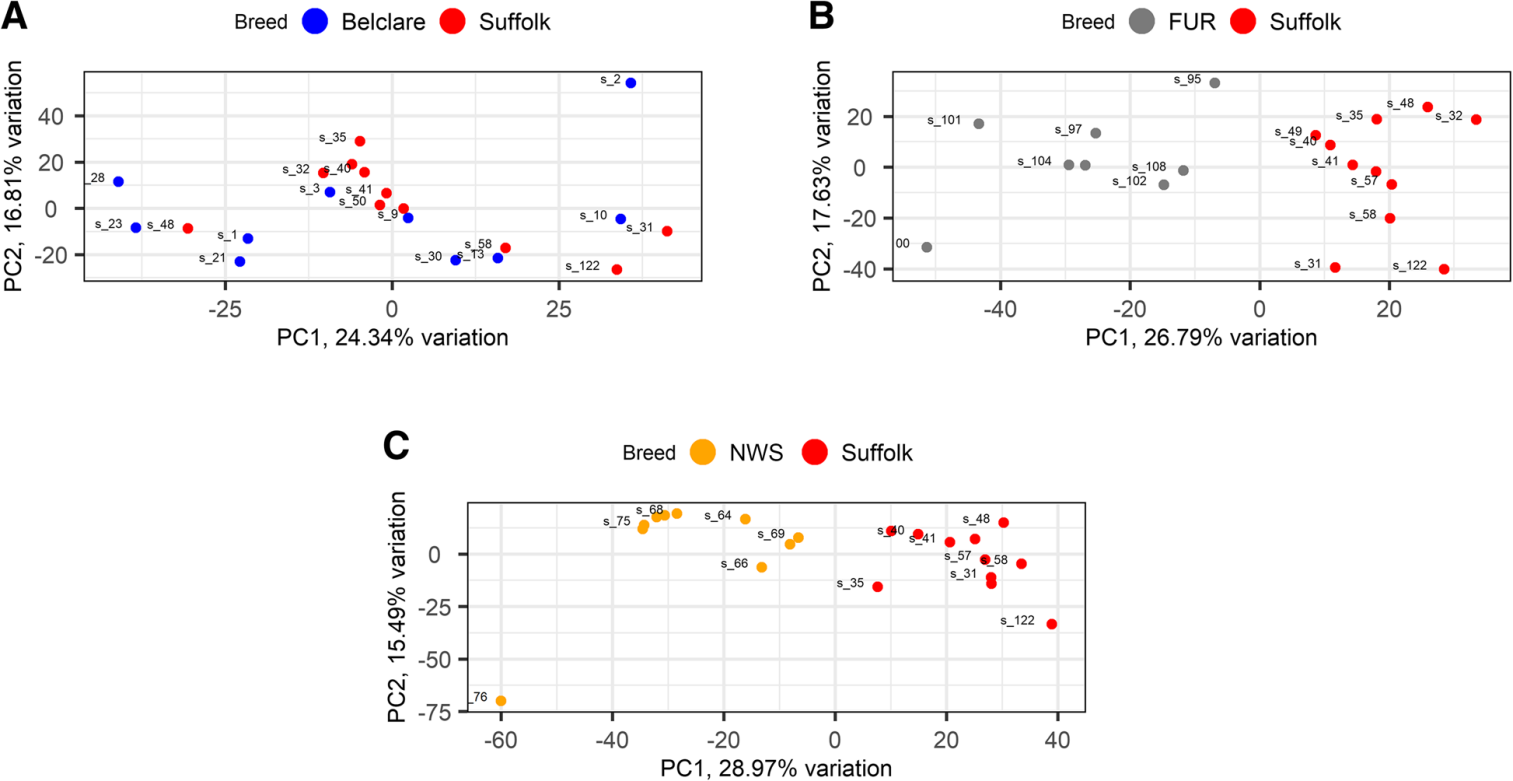
Principal component analysis (PCA) plots show distribution of RNA-sequencing samples, where colours indicate the two ewe breeds in each comparison: Belclare (**A**), Fur (**B**) and Norwegian White Sheep (NWS) (**C**) compared to Suffolk at the follicular phase of a synchronised oestrous cycle

Principal component analysis (PCA) was used to assess the distribution of cervical samples between ewe breeds known to have divergent fertility following cervical AI with frozen-thawed semen. We used the low fertility Suffolk breed as a reference level due to its lowest fertility. Figure 1 shows an evident separation between Suffolk and both Norwegian ewe breeds (NWS and Fur), although there is no clear separation between Suffolk and Belclare ewes.

### Gene expression analysis revealed greatest differences between the low fertility Suffolk breed and high fertility NWS

**Fig. 2:**
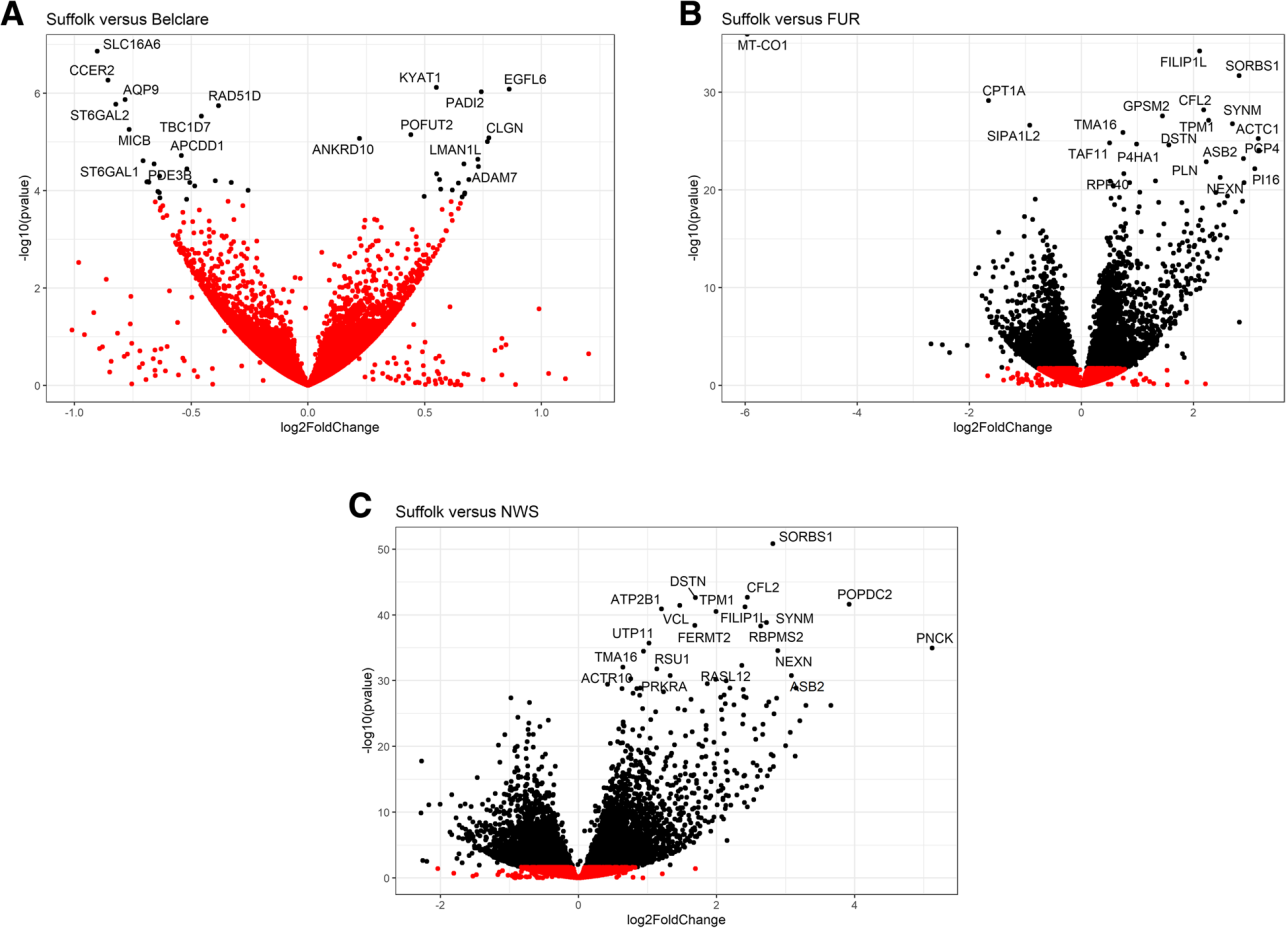
Gene expression data presented as volcano plots at the follicular phase of a synchronised oestrous cycle for Belclare (**A**), Fur (**B**) and Norwegian White Sheep (NWS) (**C**) compared to the low fertility Suffolk breed using log values of the fold change and *P*-value. Each point represents a single gene, with those in black representing genes that survived the cut off thresholds of adjusted *P* <  0.05 and FC > 1.5 and red points represent genes with a *P* > 0.05

Ewe breed was shown to significantly affect the cervical gene expression, showing extensive alterations between the low fertility Suffolk breed and the two ewe breeds with highest fertility (Fur and NWS). The identified differentially expressed genes (DEGs) were found to be significant with a *P* <  0.05 and FC > 1.5. RNA-sequencing detected 27 DEGs (14 with lower-expression and 13 with higher-expression), 1827 (890 with lower-expression and 937 with higher-expression) and 2641 (1352 with lower-expression and 1289 with higher-expression) in Belclare, Fur and NWS respectively compared to Suffolk. Ewe breed differences are evident between the highest fertility breed (NWS) and Suffolk (lowest fertility) as illustrated on the volcano plot (Fig. 2).

The top 5 DEGs with the highest expression in Suffolk compared to Belclare is shown in Supplementary Table 1. These include *SLC16A6* (Solute Carrier Family 16 Member 6), *CCER2* (Coiled-coil glutamate-rich protein 2), *AQP9* (Aquaporin 9) and *ST6GAL2* (Beta-galactoside alpha-2,6-sialyltransferases). The top 5 DEGs with the lowest expression in Suffolk compared to Belclare is also shown in Supplementary Table 1. These include *EGFL6* (EGF Like Domain Multiple 6), *CLGN* (Calmegin), *LMAN1L* (Lectin, Mannose Binding 1 Like) and *ADAM7* (ADAM Metallopeptidase Domain 7).

**Fig. 3:**
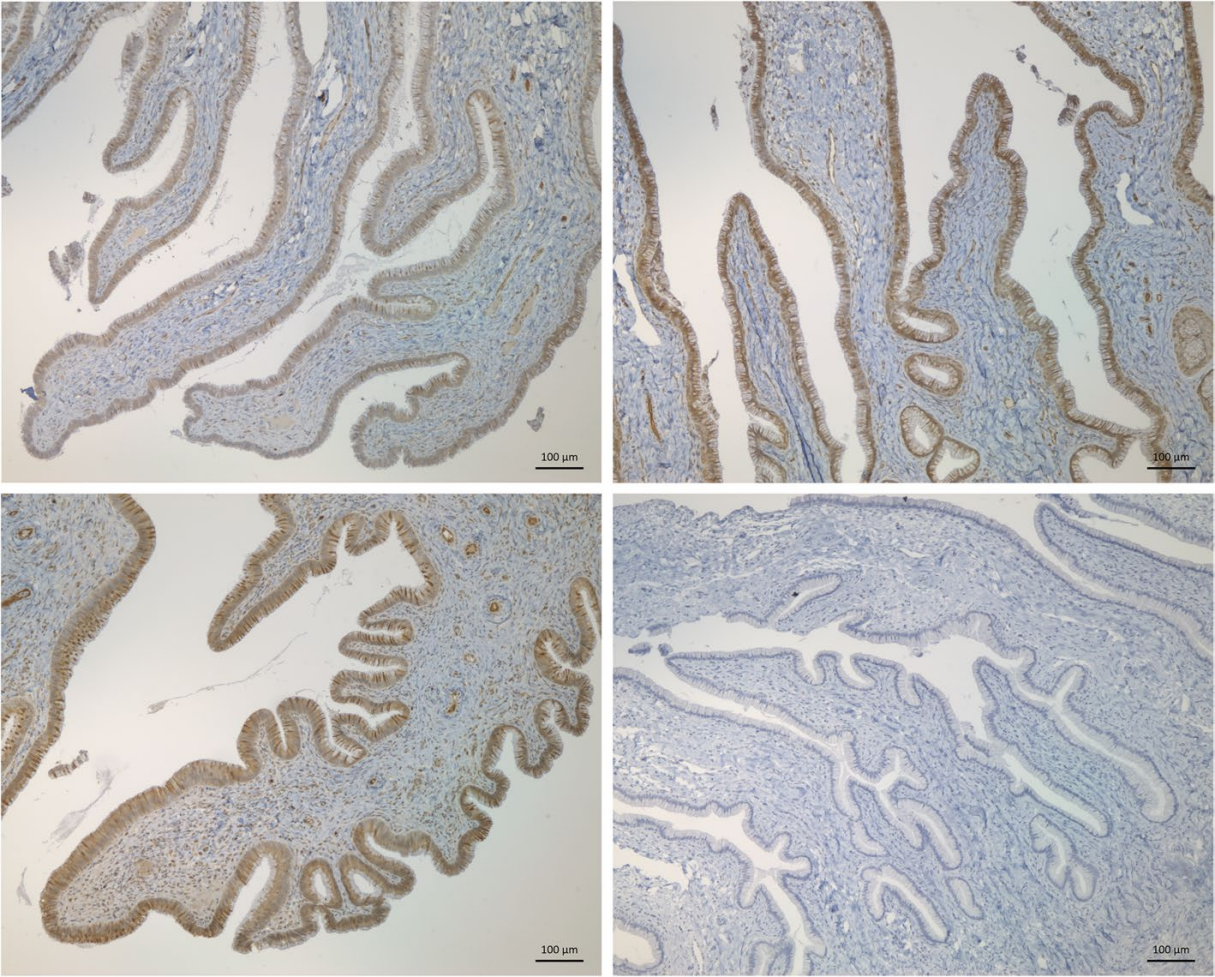
Representative images of COX-1 expression (stained brown) in cervical tissue from Norwegian White Sheep (NWS), Fur and Suffolk at the follicular phase of a synchronised oestrous cycle using immunohistochemical COX-1 staining (magnification: 100x). A representative image of a negative control (without adding antibody) is also shown

The top 5 DEGs with the highest expression in Suffolk compared to Fur ewes at the follicular phase included: Cytochrome C Oxidase Subunit 1 gene (*COX-1*), Serpin Peptidase Inhibitor (*SERPINF2*), Mucin 5 AC (*MUC5AC*; Supplementary Table 2). *COX-1* was the gene with the highest difference between Fur and Suffolk, presenting higher levels of *COX-1* in the low fertility Suffolk breed compared to both Fur (FC = 62.23; Table S6) and NWS (FC = 0.48; Table S7) (*P* <  0.05) but not Belclare. The presence of COX-1 in the cervical tissue of Suffolk and the two Norwegian breeds (Fur and NWS) was validated by COX-1 staining (Fig. 3). The top 5 DEGs with the lowest expression in Suffolk compared to Fur are also shown in Supplementary Table 2. These include genes involved in actin-filament dynamics (*ACTA1, ACTG2*), Ankyrin Repeat and SOCS Box Protein 2 (*ASB2*) and Peptidase Inhibitor 16 (*PI16*).

The top 5 of the highest expressed genes in Suffolk compared to NWS are the Forkhead Box C1 (*FOXC1*), Secretoglobin Family 2A Member 1 (*SCGB2A1*), FMS-Like Tyrosine Kinase 3 (*FLT3)* and *CD177* (Supplementary Table 3). The top 5 DEGs with the lowest expression in Suffolk compared to NWS, include genes such as Pregnancy Up-Regulated Nonubiquitous CaM Kinase (*PNCK*), Desmin (*DES*) and Actin Gamma 2, Smooth Muscle (*ACTG2*) (Supplementary Table 3). The mapping information and the full lists of the DEGs for all the three comparisons can be found in Supplementary Tables 4, 5, 6 and 7.

### Gene ontology analysis identified repressed immune response pathways in NWS compared to the low fertility Suffolk breed

Identification of enriched pathways using GO analysis revealed no subcategory with significant enrichment in Belclare compared to Suffolk ewes, although GO showed enriched pathways in Fur and NWS compared to Suffolk. The top 5 biological processes with lower and higher expression in Fur compared to Suffolk ewes are shown in Table 1. These include repressed pathways involved in multicellular organismal processes, biological adhesion and response to external stimulus in Fur compared to Suffolk (*P* <  0.05). However, induced pathways in Fur were involved in muscle contraction and circulatory system development. All the enriched pathways involved in biological processes are listed in Supplementary Tables 8 and 9.

**Table 1:** The top 5 biological processes enriched pathways with lower and higher expression in Fur (A) and Norwegian White Sheep (NWS) (B) compared to Suffolk at the follicular phase of a synchronised oestrous cycle

Term name	Term ID	*P*-value
A		
Lower in Fur compared to Suffolk		
Multicellular organismal process	GO:0032501	< 0.001
Regulation of multicellular organismal process	GO:0051239	< 0.001
Biological adhesion	GO:0022610	< 0.001
Response to external stimulus	GO:0009605	< 0.001
System development	GO:0048731	< 0.001
Higher in Fur compared to Suffolk		
Muscle contraction	GO:0006936	< 0.001
Muscle structure development	GO:0061061	< 0.001
Muscle tissue development	GO:0060537	< 0.001
Circulatory system development	GO:0072359	< 0.001
Cellular developmental process	GO:0048869	< 0.001
B		
Lower in NWS compared to Suffolk		
Immune response	GO:0006955	< 0.001
Defence response	GO:0006952	< 0.001
Response to external stimulus	GO:0009605	< 0.001
Multicellular organismal process	GO:0032501	< 0.001
Cell-cell adhesion	GO:0098609	< 0.001
Higher in NWS compared to Suffolk		
Muscle contraction	GO:0006936	< 0.001
Muscle structure development	GO:0061061	< 0.001
Muscle tissue development	GO:0060537	< 0.001
Extracellular matrix organization	GO:0030198	< 0.001
System development	GO:0048731	< 0.001

The induced enriched pathways in NWS compared to Suffolk were also related to muscle development as previously identified in Fur ewes. Other pathways such as ECM organization and system development were also induced in NWS compared to Suffolk (*P* <  0.05; Table 1). Interestingly, GO analysis revealed a repression in pathways involved in immune response, response to external stimulus and cell-cell adhesion in NWS compared to the low fertility Suffolk breed. All the enriched pathways involved in biological processes are listed in Supplementary Tables 10 and 11.

### Compromised cervical immune protection against pathogens may contribute to reduced fertility

A number of immune genes were also significantly differentially expressed between Suffolk and both Norwegian ewe breeds during the synchronised follicular phase. The majority of differentially expressed immune genes were significantly lower expressed in the Suffolk compared to the Fur, and these are spread across multiple functional classes including CD markers, Major Histocompatibility Complex (MHC), cytokines, chemokines, and genes involved in the antimicrobial response. Comparing the Fur to the Suffolk, CD markers such as *CD27* - a costimulatory molecule that regulates survival and activation of lymphocytes and *CD80* - a costimulatory molecule known for its role in T-cell activation and multiple cytokine receptors were higher. Similarly genes encoding complement proteins (*C3*), apolipoprotein E (*APOE*), the genes *IGF2* and *SAA1* were significantly higher. Of particular note, the MHC gene *BOLA3* (BolA Family Member 3), *CD274* and multiple chemokine genes including *CCL2* and *CCL27* were significantly lower.

Cytokines *IL17B, IL18, IL33, IL34* and *TGFβ* (Transforming Growth Factor Beta) had lower expression. Members of the S100 family of calcium regulated multifunctional peptides (*S100B* and *S100A10*) were similarly reduced in expression. A similar profile in DEG is evident comparing the NWS to the Suffolk but a greater number of CD receptors had higher expression in the Suffolk – including *CD14, CD27, CD79B* and *CD80. TLR8* and *TLR10* had significantly higher expression. *CCR5, CXCR4, CCR6* and *SAA1* chemokine receptors also had higher expression in the Suffolk.

Multiple members of the MHC class of molecules (*HLA-DOA, HLA-DQB1 and HLA-DRA* were also elevated in the Suffolk. In contrast, genes encoding complement protein C7 that had relatively high expression had significantly lower expression in the Suffolk compared to the NWS. Expression of the *BOLA3* gene and multiple chemokines (*CCL2, CCL11* and *CCL21*) were all similarly lower. *CD274, IGF1* as well as multiple cytokines (*IL17B, IL18, IL33, IL34* and many *TGFβ* gene family members) had all lower expression. *S100B* and *S100A10* also had lower expression in the Suffolk compared to the other ewe breeds.

### Gene co-expression analysis identified similar expression patterns between both Norwegian ewe breeds

**Fig. 4:**
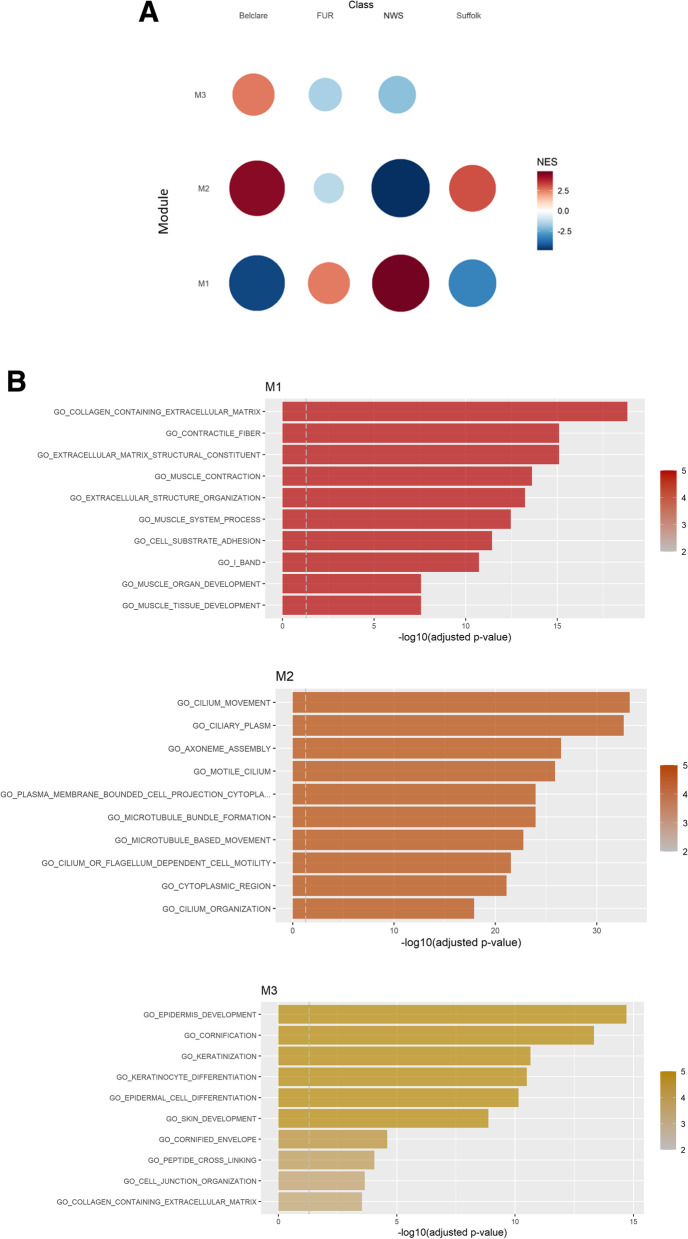
Gene co-expression analysis across the four ewe breeds (Suffolk, Belclare, Fur and NWS) at the follicular phase of a synchronised oestrous cycle. **A** Gene set enrichment analysis identified module 1, 2 and 3 to differ between Irish and Norwegian ewe breeds. **B** Over-representation analysis of genes showed the gene ontology terms in module 1, 2 and 3. The size of the circle is proportional to its normalized enriched score (NES) value. NWS = Norwegian White Sheep

Using the RNA-sequencing data of the four ewe breeds, we performed a co-expression analysis, which allowed us to identify and analyse co-expression modules. We identified five modules, from which module 1, module 2 and module 3 showed different co-expression patterns between Irish (Suffolk and Belclare) and Norwegian ewe breeds (Fur and NWS; Fig. 4). Regarding module 1, Fur and NWS had significantly enriched of pathways related to muscle contraction and ECM development, while Belclare and Suffolk ewes had lower expression of these enriched pathways (*P* <  0.05; Fig. 4). The main regulators genes of this module were *TGFβ1I1* (Transforming Growth Factor Beta 1 Induced Transcript 1), *LIMS2* (LIM Zinc Finger Domain Containing 2), *MYL9* (Myosin Light Chain 9), *TPM1* (Tropomyosin 1) and *MYLK* (Myosin Light Chain Kinase). Module 2 had enriched pathways related with cell components which were higher expressed in both Irish breeds and lower expressed in Norwegian breeds (*P* <  0.05). Regarding module 3, Irish ewe breeds had higher expression of pathways involved in keratinization and cell-cell junction and Norwegian ewe breeds lower expression (*P* <  0.05).

## Discussion

In sheep, synchronisation of oestrous to control the timing of ovulation is essential for most farmers performing AI. Here, we assessed the transcriptome of the cervix, recovered at the follicular phase of a synchronised oestrous, in ewe breeds with divergent pregnancy rates following cervical AI using frozen-thawed semen. The main findings of this study were that genes regulating muscle contraction and development were higher expressed in Fur and NWS (both with high fertility) compared to the low fertility Suffolk breed. Interestingly, the immune response pathway was lower in NWS compared to Suffolk. The differential expression of multiple informative immune genes in this dataset supports the case for a divergent immune profile between ewe breeds – particularly between the NWS and Suffolk breeds. The response between ewe breeds previously reported during the follicular phase of a natural oestrous cycle [20], is again evident in the profiles detected in this study after the use of hormones for oestrous synchronisation, leading to the conclusion that oestrous synchronisation does not seem to be the principal factor of the known ewe breed differences in fertility.

The outcome of cervical AI with frozen-thawed semen is limited by the inability of sperm to traverse the cervix of some ewe breeds. Previous studies have reported that this is due to the ewe breed used in Norway as vaginal insemination (shot-in-the-dark) with frozen-thawed semen at a natural oestrous achieves pregnancy rates in excess of 70% [2, 21]. However, the molecular mechanisms underlying ewe breed differences in sperm transport through the cervix remain unknown.

In the present study, we identified that muscle contraction (including genes involved in Actin-filament dynamics (*ACTA1, ACTA2*), Sodium Calcium Carrier (*SLC8A1*), *TPM1* (Tropomyosin 1), *MYLK* (Myosin Light Chain Kinase), etc.) and ECM development pathways (including genes such as *TGFβ1I1*, ADAM Metallopeptidases, Matrix Metallopeptidase *MMP3* and genes involved in collagen organization (*COL6A3*, *COL4A6*, *COL4A5*)) were increased in Fur and NWS compared to Suffolk. The extracellular matrix and the smooth muscle of the cervix undergoes to a cyclic remodelling influenced by changes in hormonal levels [22]. High levels of oestrogen around the time of ovulation regulates oxytocin production and increases the contractions of the cervix by increasing intracellular calcium levels through sodium calcium carriers [23]. As reported by Kershaw et al. 2007, [24] oxytocin also stimulates the production of PGE_2_ via prostaglandin endoperoxide synthase 2 (PTGS2 or COX-2), which results in cervical softening and myometrial contractions. All the evidence suggests that smooth muscle contractions of the cervix play an important role in cervical sperm transport [25, 26] and these contractions increase at the follicular phase of the oestrous cycle [25]. A previous study reported that cervical contractions of the sheep cervix were increased at oestrous compared to the luteal phase at both types of oestrous (synchronised versus natural). These studies indicate that oestrous synchronisation protocols have no effect on cervical contractility as there were no differences between a synchronised and a natural oestrous cycle [27]. However, other factors such as differences in the microbiome could also affect cervical contractility as reported by Lee et al. 2020 [28], which showed reduced muscle contraction in the presence of *Chlamydia* infection. The differences in genes relating to cervical contractility between the breeds need to be taken in the context that the differences in cervical sperm transport between ewe breeds is most evident with frozen-thawed semen [3, 29, 30]. Thus, cervical contractility alone would unlikely explain the differences in pregnancy rates.

*COX-1* was the gene with the highest difference between Fur and Suffolk, presenting higher levels of *COX-1* in the low fertility Suffolk breed. In addition, *COX-1* was also higher in Suffolk compared to NWS (highest fertility) and this trend was also clearly evident when assessed using immunohistochemical COX-1 staining (Fig. 3). COX-1 is an enzyme widely expressed in most tissues including gastrointestinal mucosa, platelets, endothelium, kidneys and the uterus [31]. Arachidonic acid released from neighbouring damaged membranes is converted by COX-1 and COX-2 into prostaglandins. COX-1 is principally involved in tissue homeostasis and while levels are typically stable, elevated levels can be induced under stressful conditions [32]. Therefore, the higher expression of COX-1 observed in the cervix of the low fertility Suffolk breed could reflect a sub-optimal environment and initial stage of inflammation produced by pathogens that potentially affects sperm migration through the cervix.

Our results revealed DEGs encoding for cellular and immune receptors (CD receptors and TLRs) involved in pathogen detection that were significantly different between NWS and Suffolk. For example, levels of *CD80* were higher in Suffolk compared to NWS, which could be related to the presence of an altered microbiome in the low fertility Suffolk breed. Agrawal et al. 2008 [33] identified higher expression on *CD80* in the cervix of women with *Chlamydia* infection, which were correlated with fertility disorders. They concluded that the role of CD80 activation in the secretion of cytokines could be the main mechanism which decides whether the infection is cleared or will produce a pathological response with the latter response the most likely in the cervix of the low fertility Suffolk breed. Toll-like receptors that are also essential for the initial detection and response to pathogens were detected in this study. Specifically, *TLR8* and *TLR10* had lower expressed in NWS compared to Suffolk. *TLR8* has been previously detected throughout the female reproductive tract, suggesting a constant antibacterial function throughout the female reproductive tract [34]. It has been described that TLR8 recognise Gram-positive pyogenic bacteria such as *Staphylococcus aureus* and group *B streptococcus,* suggesting that the cervix of Suffolk ewes could be having an active immune response against these species of bacteria [35]. Therefore, the reduced immune response in the cervix of the high fertility ewe breeds could be affording sperm safer passage through the cervix, avoiding female immune cells and thereby reaching the site of fertilisation. A number of studies have reported that seminal plasma regulates sperm movement capacitation [36] and the acceptance of sperm by the female reproductive tract [37]. Proteins are a major component of seminal plasma and signalling factors along with glycoproteins are intrinsically involved in sperm-binding properties to the female reproductive tract [38] as well as acting as the main modulator of sperm function [36]. In addition, it has been reported that freshly ejaculated and frozen-thawed sperm had less binding to polymorphonuclear neutrophils compared to epididymal sperm in vitro [39]. However, the neutrophil binding mechanism of action in sheep has not been elucidated. The increase in inflammation evident in the cervix of the low fertility Suffolk breed could be associated with an increase in neutrophils. This has implications on the rate of removal of pathogens as well as damaged sperm while allowing a small elite population of sperm to traverse the cervix.

Major histocompatibility complex receptors involved in antigen presentation by innate cells were also differentially expressed between high and low fertility ewe breeds. The expression of the complement *C3* was lower in Suffolk compared to Fur ewes. Previous studies have detected the expression of C components in human cervical epithelial cells [40] and in cervical mucus from sheep [17, 41]. The complement components help to fight off infections [42] and deficiencies in C3 in the cervix have been shown to lead to severe infections [40], which could be the case of the Suffolk breed. Cervical signalling molecules such as chemokines had different profiles of expression between high and low fertility breeds. CC-chemokine ligand 2 (*CCL2*) has been previously detected in higher amounts in the cervical mucus compared to uterine secretions [43]. Oestrogens decrease the secretion of *CCL2* from the uterine fibroblasts, but not from the cervical fibroblasts [44]. Both types of fibroblasts secrete CCL2 in response to pathogens so as to alert immune cells and recruit them to the sites of infection [45]. We identified higher expression of *CCL2* in the high fertility ewe breeds (Fur and NWS) compared to Suffolk suggesting that fibroblast immune protection against viruses is enhanced in the high fertility breeds. Chemokines receptors such as CCR5 have been identified in higher levels in women with *Chlamydial* infection compared to uninfected women, which was also linked with higher susceptibility of immunodeficiency virus infection since T-cells with CCR5 are the main targets cells of this virus [46]. In our study, there were higher levels of *CCR5* in Suffolk compared to NWS, which suggest that the cervix of the low fertility Suffolk breed could be more susceptible to co-infections that can be heightened by the process of insemination and the introduction of pathogens to the female reproductive tracts, resulting in a higher immune response against sperm since sperm survival is achieved by decreasing the antigen-presenting capacity of the dendritic cells, monocytes, and macrophages and/or blocking NK cells as well as T and B-cells against sperm immune regulatory biomolecules in the female reproductive tract [47]. The heightened expression of the *S100* genes in both Norwegian breeds, which are known to have an antimicrobial role [48] suggest that the protection of the cervix may be compromised in the Irish breeds as we have previously reported at the follicular phase of a natural oestrous cycle.

## Conclusions

In conclusion, this study provides for the first time, data on the cervical transcriptome profile of four ewe breeds with divergent pregnancy rates following cervical AI using frozen-thawed semen at the follicular phase of a synchronised oestrous cycle. We identified evident differences in muscle contraction and immune response pathways. The expression of genes encoding effector molecules (cytokines and S100 genes), signalling molecules (chemokines), cellular and immune receptors (CD receptors and TLRs) and MHC receptors all signifying an active and distinct immune response in the cervix. Further investigation is needed in order to confirm if specific markers of an active immune response could affect the integrity of the cervical barrier or impaired cervical sperm transport.

## Methods

### Ethical approval

Protocols were developed in accordance with the Cruelty to Animals Act (Ireland 1876, as amended by European Communities regulations 2002 and 2005) and the European Community Directive 86/609/EC. In Norway the study was approved by Norwegian Food Safety Authority (FOTS ID 13168). In Ireland, all animal procedures were conducted under experimental license from the Health Products Regulatory Authority and the study was approved by the Teagasc animal ethics committee. This study was carried out in compliance with the ARRIVE Guidelines for reporting animal research [49].

### Experimental design and tissue collection

The animal model has previously been described by Abril-Parreño et al. 2021a [7]. This experiment was performed as a part of larger study, which aimed to interrogate the ewe breed effect on mucus properties and anatomical characteristics across the oestrous cycle at both a synchronised and a natural oestrous. In this study, we interrogated the gene expression of the sheep cervix of four ewe breeds across two countries: Ireland (Belclare and Suffolk; medium and low fertility, respectively) and Norway (NWS and Fur; both with high fertility compared to the Irish ewe breeds) at the follicular phase of a synchronised oestrous cycle. We used these ewe breeds due to their known different pregnancy rates following cervical/vaginal AI with frozen-thawed semen. Suffolk ewes were the reference level (negative control) in this analysis as they have the lowest pregnancy rates reported [3]. All the ewes used in this study were multiparous in the range of 4 to 5 years old with an average live weight of 79.3 ± 2.38, 65.6 ± 3.47, 80.9 ± 3.36 and 82.1 ± 2.46 kg for Belclare, Fur, NWS and Suffolk, respectively. Post-mortem cervical tissue samples were collected from the four ewe breeds at the follicular phase of a synchronised cycle (*n* = 10, 8, 10 and 11 Belclare, Fur, NWS and Suffolk ewes, respectively). The ewes were synchronised using the protocol supplied in Additional file 4. Following euthanasia, the ovaries were assessed for the presence or absence of dominant follicles and/or a fresh ovulation as evident by a corpus hemorrhagicum (follicular phase). The reproductive tracts were then longitudinally opened and two sections were taken from the mid-region of the cervix while avoiding the cervical folds. All samples were snap-frozen in liquid nitrogen, and subsequently stored at − 80 °C until RNA isolation. To perform the immunohistochemical staining another cervical tissue section was taken and immersed in formalin until further tissue processing.

### Tissue processing and RNA extraction

In order to lyse the tissue and extract the RNA, frozen cervical tissue was immersed in TRIzol reagent and then homogenized using the homogenizer (Bio-gen Pro200 Homogenizer, Pro Scientific). The RNA extraction was completed using the RNeasy Kit (Quiagen Ltd., Crawley, West Sussex, UK) according to the manufacturer’s instructions. Total RNA concentration was quantified using the Nanodrop ND-1000 UV-Vis Spectophotometer (NanoDrop Technologies Inc., Wilmington, DE, USA). Quality of RNA was ascertained with the use of 2100 Agilent Bioanalyzer (Agilent Technologies, Santa Clara, CA, USA). RNA integrity number (RIN) was greater than 7 in all samples and RNA aliquots were frozen at − 80 °C after extraction.

### Library preparation and RNA-sequencing

Illumina® TruSeq® Stranded mRNA Library preparation Kit RNA libraries was used to prepare 39 RNA samples. Indexes were allocated to specific samples prior to library construction so that each sample within a pool had a unique bar code. Following adapter ligation, DNA fragments were selectively enriched by performing PCR. Quality control checks were performed to assess the quality and quantity of the ds cDNA libraries. The Agilent 2100 Bioanalyzer (Agilent Technologies) was used to assess purity of the samples, using the Agilent DNA 1000 kit. Library quantity was measured using the Qubit fluorometer. These steps were previously reported by Brewer et al. 2020 [50]. All libraries were sequenced on an Illumina NovaSeq sequencer by Macrogen, Inc. (Seoul, Republic of Korea) where they were sequenced using an Illumina NovaSeq. Sequencing was performed for each sample at 2 × 150 bp paired end reads (50 M reads) as previously described [20].

### Differential expression analysis

Quality assessment of the raw sequence data was carried out using the software FastQC (v 0.11.8; http://www.bioinformatics.babraham.ac.uk/projects/fastqc/). Data were quality and adapter trimmed using the BBDuk java package to trim Illumina adapter sequences and any low quality bases (Phred score < 20) from the 3′ end of sequence read pairs. Reads were aligned to the ovine genome Oar_v3.1 using the Spliced Transcripts Alignment to a Reference (STAR) aligner. A maximum of two mismatches with the reference genome were allowed and only uniquely mapped read pairs were retained for downstream analysis. Read counts overlapping all protein coding genes in the Oar_v3.1 Ensembl (v.95) annotation were estimated using featureCounts. To filter out lowly expressed genes, genes with less than one count per million in at least 10 samples were discarded from the analysis. Remaining gene counts were normalized using the median of ratios method as implemented in DeSeq2 (version 1.130.0) [51] to account for varying sequencing depth between samples. Transcript counts were modelled by fitting the data to a negative binomial distribution using genewise dispersion estimates and DEGs were identified with a generalized linear model likelihood ratio test. Statistical tests were corrected for multiple testing using the Benjamini-Hochberg method. Only DEGs with an adjusted *P* <  0.05 and a FC threshold of 1.5 were used for further differentially expressed gene data exploration and pathway analysis.

### Functional and pathway enrichment analysis

Aggregated functional profiles of genes and gene clusters in the DEGs lists were identified using the gProfiler2 (v. 0.2.0) package. GO terms and Reactome pathways were analysed with an enrichment threshold cut-off of *P* <  0.05. The R package rrvgo (v.1.1.4) was used to reduce the redundancy of significantly enriched GO terms by grouping similar terms based on their similarity within the GO hierarchy. Gene co-expression network analyses was carried out using the R package Cemitools (v1.14.0). For any modules identified a gene set enrichment analysis was carried out to indicate if each module was induced or repressed in the different ewe breeds. Finally, an over representation analysis was used to identify in each module enriched biological functions.

### Immunohistochemistry preparation

Immunohistochemistry staining for COX-1 was performed on cervical tissue of a subset of Norwegian White Sheep (NWS), Fur and Suffolk using an avidin-biotin-peroxidase method (Vectastain Elite ABC-HRP Kit, Peroxidase (Rabbit IgG, PK-6101), Vector Laboratories USA). Formalin-fixed cervical biopsies were paraffin-embedded, sectioned at 3 μm thickness, rehydrated in graded ethanol, and demasked in a microwave oven 15 minutes 121 °C in 0.01 M citrate buffer (pH 6.0). Nonspecific endogenous peroxidase activity was blocked by treatment with 1% hydrogen peroxide for 10 minutes. The sections were exposed to 2% normal goat serum for 20 minutes at room temperature, before incubation with the primary antibody overnight at 4 °C. The primary antibody was diluted in 1% bovine serum albumin in Tris-buffered saline medium. The rabbit polyclonal Cyclooxygenase-1/COX-1 antibody (abcam, ab244261) was diluted 1:100. The secondary antibody, biotinylated goat anti-rabbit (dilution 1:200) was incubated for 30 minutes at room temperature before ABC complex was added and incubated for 30 minutes. The immunoreaction was visualized using the chromagen 3, 3-diaminobenzidine tetrahydrochloride (DAB, Sigma Chemical Co.) and contrast staining with Mayer’s hematoxylin. Immunohistochemistry was performed in the absence of the primary antibody as a negative control.

## Supplementary Information

**Additional file 1: Table S1.** Top 5 differentially expressed genes (withhigher and lowerexpression) in Suffolk compared to Belclare. The genes shown in these tables were found to be significant with a *P* <  0.05 and FC > 1.5. **Table S2.** Top 5 differentially expressed genes (with higher and lower expression) in Suffolk compared to Fur. The genes shown in these tables were found to be significant with a *P* <  0.05 and FC > 1.5.**Table S3.** Top 5 differentially expressed genes (with higher and lower expression) in Suffolk compared to Norwegian White Sheep (NWS). The genes shown in these tables were found to be significant with a *P* <  0.05 and FC > 1.5.

**Additional file 2: Table S4.** Mapping information. **Table S5.** Differentially expressed genes (DEGs) in Suffolk compared to Belclare. **Table S6.** Differentially expressed genes (DEGs) in Suffolk compared to Fur. **Table S7.** Differentially expressed genes (DEGs) in Suffolk compared to Norwegian White Sheep (NWS).

**Additional file 3: Table S8.** List of biological processes with higher expression in Fur compared to Suffolk. **Table S9.** List of biological processes with lower expression in Fur compared to Suffolk. **Table S10.** List of biological processes with higher expression in Norwegian White Sheep (NWS) compared to Suffolk. **Table S11.** List of biological processes with lower expression in Norwegian White Sheep (NWS) compared Suffolk.

**Additional file 4.** Protocol for oestrous synchronisation.

## Data Availability

The datasets generated and/or analysed during the current study are available in the NCBI Gene Expression Omnibus https://www.ncbi.nlm.nih.gov/geo/ under accession number GSE179486.

## Abbreviations

AI: Artificial Insemination
DEG: Differentially Expressed Gene
ECM: Extracellular Matrix
FC: Fold Change
GO: Gene Ontology
NWS: Norwegian White Sheep
PCA: Principal Component Analysis
PCR: Polymerase Chain Reaction
RIN: RNA Integrity Number
STAR: Spliced Transcripts Alignment to a Reference
