# Supplementary material for: Ewe breed differences in the cervical transcriptome at the follicular phase of a synchronised oestrous cycle
Source: BMC Genomics. 2022 May 11;23:363. doi: 10.1186/s12864-022-08603-8 (PMC9097332; doi:10.1186/s12864-022-08603-8)
Supplement: Supplementary file 1 — Additional file 1: Table S1. Top 5 differentially expressed genes (withhigher and lowerexpression) in Suffolk compared to Belclare. The genes shown in these tables were found to be significant with a P < 0.05 and FC > 1.5. Table S2. Top 5 differentially expressed genes (with higher and lower expression) in Suffolk compared to Fur. The genes shown in these tables were found to be significant with a P < 0.05 and FC > 1.5.Table S3. Top 5 differentially expressed genes (with higher and lower expression) in Suffolk compared to Norwegian White Sheep (NWS). The genes shown in these tables were found to be significant with a P < 0.05 and FC > 1.5. [file 12864_2022_8603_MOESM1_ESM.docx]

**Additional File 1: Ewe breed differences in the cervical transcriptome at the follicular phase of a synchronised oestrous cycle**

**Table S1**. Top 5 differentially expressed genes (with higher and lower expression) in Suffolk compared to Belclare. The genes shown in these tables were found to be significant with a *P < 0.05* and FC > 1.5.

| Gene symbol | *P*-value | | FC | Ensembl ID | Gene name | Gene function |
| --- | --- | --- | --- | --- | --- | --- |
|  | |  | Higher in Suffolk compared to Belclare | | | |
| *SLC16A6* | 0.002 | | 1.86 | ENSOARG00000015264 | Solute Carrier Family 16 Member 6 | -Transport across the plasma membrane of monocarboxylates branched-chain oxo acids derived and ketone bodies |
| *CCER2* | 0.003 | | 1.81 | ENSOARG00000005905 | Coiled-Coil Glutamate Rich Protein 2 | -Extracellular component  - Protein binding |
| *ST6GAL2* | 0.003 | | 1.76 | ENSOARG00000020956 | Beta-galactoside alpha-2,6-sialyltransferases | - Transfer sialic acid with an α-2,6-linkage to the Gal-β-1,4-GlcNAc glycan chain |
| *AQP9* | 0.003 | | 1.72 | ENSOARG00000020858 | Aquaporin 9 | -Water channel  -Mediates passage of a wide variety of small, non-charged solutes |
| *MICB* | 0.008 | | 1.70 | ENSOARG00000009868 | MHC class I polypeptide-related sequence B | - Antigen presentation  - Belongs to the MHC class I family  - Acts as a stress-induced self-antigen that is recognized by gamma delta T cells |
|  | |  | Lower in Suffolk compared to Belclare | | | |
| *EGFL6* | 0.003 | | -1.81 | ENSOARG00000011304 | EGF Like Domain Multiple 6 | - Promotes matrix assembly  - Bind integrin α-8/β-1 |
| *CLGN* | 0.009 | | -1.71 | ENSOARG00000012717 | Calmegin | - Calcium ion and protein binding  - Binding of sperm to zona pellucida |
| *LMAN1L* | 0.010 | | -1.70 | ENSOARG00000003153 | Lectin, Mannose Binding 1 Like | -Transport of newly synthesized glycoproteins |
| *PADI2* | 0.003 | | -1.67 | ENSOARG00000011146 | Peptidyl Arginine Deiminase 2 | -Catalyzes the deamination of arginine residues of proteins |
| *ADAM7* | 0.023 | | -1.65 | ENSOARG00000009977 | ADAM Metallopeptidase Domain 7 | -Proteolysis  -Sperm binding protein |

**Table S2**. Top 5 differentially expressed genes with higher and lower expression) in Suffolk compared to Fur. The genes shown in these tables were found to be significant with a *P < 0.05* and FC > 1.5.

| Gene symbol | *P*- value | | FC | | Ensembl ID | Gene name | Gene function |
| --- | --- | --- | --- | --- | --- | --- | --- |
|  | |  | | Higher in Suffolk compared to Fur | | | |
| *COX-1* | 0.000 | | 62.23 | | ENSOARG00000000016 | Mitochondrially Encoded Cytochrome C Oxidase I | - Component of the cytochrome c oxidase, which drives oxidative phosphorylation.  - Enzyme in the synthesis of prostaglandins and COX inhibitors have been shown to have an effect on female fertility. |
| *SERPINF2* | 0.000 | | 6.42 | | ENSOARG00000014081 | Serpin Family F Member 2 | - Serine Protease inhibitor |
| *MUC5AC* | 0.003 | | 5.11 | | ENSOARG00000009361 | Mucin 5AC, Oligomeric Mucus/Gel-Forming | - Gel-forming mucin that protects the mucosa from infection and chemical damage |
| Novel gene | 0.001 | | 4.09 | | ENSOARG00000016098 | - | - |
| *ATP8A2* | 0.000 | | 3.69 | | ENSOARG00000013009 | ATPase Phospholipid Transporting 8A2 | - Catalyzes the hydrolysis of ATP coupled to the transport of aminophospholipids from the outer to the inner leaflet of various membranes |
|  | |  | | Lower in Suffolk compared to Fur | | | |
| *PCP4* | 0.000 | | -8.96 | | ENSOARG00000009912 | Purkinje Cell Protein 4 | - Calcium-binding by calmodulin |
| *ACTC1* | 0.000 | | -8.90 | | ENSOARG00000019993 | Actin Alpha Cardiac Muscle 1 | - Actin filament-based movement |
| *PI16* | 0.000 | | -8.52 | | ENSOARG00000014123 | Peptidase Inhibitor 16 | - Peptidase inhibitor activity |
| *ACTG2* | 0.000 | | -7.47 | | ENSOARG00000012108 | Actin Gamma 2, Smooth Muscle | - Constituent of muscle tissues  - Muscle contraction |
| *ASB2* | 0.000 | | -7.42 | | ENSOARG00000014168 | Ankyrin Repeat And SOCS Box Containing 2 | -Ubiquitination and subsequent proteasomal degradation of target proteins |

**Table S3**. Top 5 differentially expressed genes (with higher and lower expression) in Suffolk compared to Norwegian White Sheep (NWS). The genes shown in these tables were found to be significant with a *P < 0.05* and FC > 1.5.

| Gene symbol | *P*-value | | FC | | Ensembl ID | Gene name | Gene function |
| --- | --- | --- | --- | --- | --- | --- | --- |
|  | |  | | Higher in Suffolk compared to NWS | | | |
| *TRPM1* | 2.97E-09 | | 4.85 | | ENSOARG00000016239 | Transient Receptor Potential Cation Channel Subfamily M Member 1 | -Calcium ion transmembrane transport |
| *FOXC1* | 1.76E-16 | | 4.83 | | ENSOARG00000002778 | Forkhead Box C1 | -DNA-binding transcriptional factor |
| *CD177* | 0.009 | | 4.58 | | ENSOARG00000008513 | CD177 Molecule | -Activation of TNF*α* primed neutrophils including degranulation and superoxide production  - Neutrophil recruitment caused |
| *SCGB2A1* | 2.33E-10 | | 4.49 | | ENSOARG00000014805 | Secretoglobin Family 2A Member 1 | - Bind androgens and other steroids |
| *FLT3* | 1.23E-06 | | 3.63 | | ENSOARG00000012379 | Fms Related Receptor Tyrosine Kinase 3 | - Cell-surface receptor for the cytokine *FLT3LG*  -Cellular response to cytokine stimulus |
|  | |  | | Lower in Suffolk compared to NWS | | | |
| *PNCK* | 1.29E-32 | | -34.78 | | ENSOARG00000007294 | Pregnancy Up-Regulated Nonubiquitous CaM Kinase | -Calcium/calmodulin-dependent protein kinase belonging to a proposed calcium-triggered signalling cascade |
| *POPDC2* | 8.62E-39 | | -15.14 | | ENSOARG00000019786 | Popeye Domain Containing 2 | - Increasing cell surface expression of the potassium channel KCNK2  -Muscle contraction |
| *PCP4* | 1.90E-24 | | -12.60 | | ENSOARG00000009912 | Purkinje Cell Protein 4 | - Calcium-binding by calmodulin |
| *ACTG2* | 1.82E-24 | | -9.82 | | ENSOARG00000012108 | Actin Gamma 2, Smooth Muscle | - Constituent of muscle tissues and muscle contraction |
| *DES* | 2.91E-22 | | -9.23 | | ENSOARG00000020185 | Desmin | - Muscle-specific type III intermediate filament essential for proper muscular function |
