# Supplementary material for: Ewe breed differences in the cervical transcriptome at the follicular phase of a synchronised oestrous cycle
Source: BMC Genomics. 2022 May 11;23:363. doi: 10.1186/s12864-022-08603-8 (PMC9097332; doi:10.1186/s12864-022-08603-8)
Supplement: Supplementary file 4 — Additional file 4. Protocol for oestrous synchronisation. [file 12864_2022_8603_MOESM4_ESM.docx]

**Additional File 4: Ewe breed differences in the cervical transcriptome at the follicular phase of a synchronised oestrous cycle**

**Protocol for oestrous synchronisation**

Estrous cycles of multiparous ewes were synchronized using intravaginal progestagen vaginal sponges (20 mg Flugestone Acetate; Chronogest® vaginal sponges, Intervet, Boxmeer, The Netherlands). After 14 days, the sponges were removed and ewes were administered equine chorionic gonadotropin (400 IU; Intervet, Boxmeer, The Netherlands). Cervical tissue was collected at 54-56 hours post sponge removal (follicular phase). Following slaughter, the ovaries were assessed for the presence or absence of dominant follicles and for evidence of a fresh ovulation to confirm the phase of the cycle (follicular phase).
